# Effects of local treatment with and without sensorimotor and balance exercise in individuals with neck pain: protocol for a randomized controlled trial

**DOI:** 10.1186/s12891-018-1964-3

**Published:** 2018-02-13

**Authors:** Munlika Sremakaew, Gwendolen Jull, Julia Treleaven, Marco Barbero, Deborah Falla, Sureeporn Uthaikhup

**Affiliations:** 1 Department of Physical Therapy, Faculty of Associated Medical Sciences, Chiang Mai University, Chiang Mai, Thailand; 2 Physiotherapy, School of Health and Rehabilitation Sciences, The University of Queensland, St Lucia, Brisbane, Australia; 3 Rehabilitation Research Laboratory 2rLab, Department of Business Economics Health and Social Care, University of Applied Sciences and Arts of Southern Switzerland, Manno, Switzerland; 4 Centre of Precision Rehabilitation for Spinal Pain (CPR Spine), School of Sport, Exercise and Rehabilitation Sciences, College of Life and Environmental Sciences, University of Birmingham, Birmingham, UK

**Keywords:** Balance, Exercises, Manual therapy, Neck pain, Sensorimotor control

## Abstract

**Background:**

Impaired cervical joint position sense and balance are associated with neck pain. Specific therapeutic exercise and manual therapy are effective for improving neck pain and functional ability but their effects on joint position sense and balance impairments remain uncertain. Changes in the joint position sense and balance may need to be addressed specifically. The primary objective is to investigate the most effective interventions to improve impaired cervical joint position sense and balance in individuals with neck pain. The secondary objective is to assess the effectiveness of the interventions on pain intensity and disability, pain location, dizziness symptoms, cervical range of motion, gait speed, functional ability, treatment satisfaction and quality of life.

**Methods:**

A 2 × 2 factorial, single blind RCT with immediate, short- and long-term follow-ups. One hundred and sixty eight participants with neck pain with impaired joint position sense and balance will be recruited into the trial. Participants will be randomly allocated to one of four intervention groups: i) local neck treatment, ii) local treatment plus tailored sensorimotor exercises, iii) local treatment plus balance exercises, and iv) local treatment plus sensorimotor and balance exercises. Participants receive two treatments for 6 weeks. Primary outcomes are postural sway and cervical joint position error. Secondary outcomes include gait speed, dizziness intensity, neck pain intensity, neck disability, pain extent and location, cervical range of motion, functional ability, perceived benefit, and quality of life. Assessment will be measured at baseline, immediately after treatment and at 3, 6, 12 month-follow ups.

**Discussion:**

Neck pain is one of the major causes of disability. Effective treatment must address not only the symptoms but the dysfunctions associated with neck pain. This trial will evaluate the effectiveness of interventions for individuals with neck pain with impaired cervical joint position sense and balance. This trial will impact on clinical practice by providing evidence towards optimal and efficient management.

**Trial registration:**

ClinicalTrials.gov (NCT03149302). May 10, 2017.

## Background

Neck pain is a common musculoskeletal disorder and a costly public health issue [1, 2]. The pain is often persistent or recurrent in nature [3, 4]. The underlying mechanisms for recurrence or persistence remain unclear but could be associated with altered proprioception from the neck muscles, which have a vital and unique role in providing input for cervical joint position sense, head and eye movement control and postural stability [5–7]. Patients with neck pain not uncommonly experience symptoms of dizziness/light headedness and unsteadiness [8, 9]. Such patients usually have impaired proprioception (cervical joint position sense) and postural instability which account for these symptoms [10–13]. These impairments can lead to decreased physical performance and increased concerns of falling, particularly the elderly [14, 15]. Further, dizziness and unsteadiness have been shown to be predictors of both poorer recovery [16] and poorer response to musculoskeletal treatment [17–19]. Thus it is important to address such symptoms and disturbances in patients with neck pain not only to gain symptomatic relief but also to reverse the impairments to improve physical performance and function.

Evidence suggests that conventional treatment of manual therapy and specific therapeutic exercise directed towards neuromuscular impairments are effective interventions for relieving neck pain [20, 21] and dizziness symptoms [22, 23] and they improve cervical joint mobility and neck muscle performance [24–26]. However, these interventions are not specifically directed towards impaired cervical proprioception and balance. The effects of exercise and manual therapy on proprioception (joint reposition sense) and balance remain uncertain [23, 24, 27].

It is recommended that changes in cervical joint position sense and balance are addressed to optimize outcomes [23, 28]. There is preliminary evidence to suggest that sensorimotor training can improve impaired cervical joint position sense [29–32], but it is unknown if sensorimotor training can improve balance or indeed if balance training can resolve impairments in cervical joint position sense. Is one, both or neither training required in addition to conventional treatment to treat patients with neck pain and proprioceptive and balance disturbances? This is an important question as it is necessary to not only understand treatment effects but also to develop the most efficient treatment strategies.

This is a mechanistic randomized clinical trial in which the effects of treatment on measures of balance and cervical proprioception will be examined. This trial will evaluate the short- and long-term benefits of conventional local treatment to the neck with and without additional sensorimotor control and balance exercise approaches for people with chronic neck pain. The primary objective is to investigate the most effective and efficient interventions to improve disturbances in cervical joint position sense and balance. More specifically, we will test if local treatment to the neck (manual therapy and therapeutic exercise) is sufficient, or whether the addition of sensorimotor control and balance exercise has a superior effect. As it is unclear whether sensorimotor control exercises will automatically improve balance and vice versa, we will also test their effects separately as well as collectively. To achieve our objective, we will test four treatment groups: (i) local neck treatment alone, (ii) local treatment plus tailored sensorimotor control exercises (joint position sense and oculomotor control), (iii) local treatment plus balance exercises and (iv) local treatment plus both sensorimotor control and balance exercises. The secondary objective is to assess the effectiveness of the interventions on neck pain intensity, neck disability, pain extent and location, any dizziness symptoms, cervical range of motion, gait speed, functional ability, treatment satisfaction and quality of life.

### Study hypotheses

Primary hypothesis: Specific training of impairments in sensorimotor control and balance will be superior for improving impairments in neck proprioception and balance.

Secondary hypothesis: Specific training of impairments in sensorimotor control and balance will be superior for reducing dizziness intensity and increasing gait speed. There will be no differences between intervention groups in outcomes of neck pain intensity, neck disability, pain extent and location, cervical range of motion, functional ability, treatment satisfaction and quality of life.

## Methods/design

### Trial design

The trial utilizes a single blind 2 × 2 factorial design, and conforms to the CONSORT recommendations [33].

### Participants

A sample of 168 women and men aged 18 years and older with neck pain and JPS and balance impairments will be recruited from the communities in Chiang Mai province, Thailand by advertising through community centers, radio, and Facebook, placing posters in hospitals, physiotherapy clinics and universities, and using our database of participants with neck pain who participated in previous studies and have given consent for future contact. Participants who respond to the advertisements will be screened by a research assistant via telephone interview. They will be potentially eligible for the trial if they meet the eligibility criteria (Table 1).

**Table 1:** Inclusion and exclusion criteria

*Inclusion criteria*
- Aged ≥18 years
- Insidious neck pain for at least 3 months
- Average neck pain intensity over the past week ≥30 mm (100 mm visual analogue scale)
- Score ≥ 10/100 on the Neck Disability Index-Thai version (NDI-TH) [47]
*Exclusion criteria*
- Previous history of neck and head trauma or surgery
- Known or suspected vestibular pathology, vertigo or dizziness from ear or brain disorders, sensory nerve pathways (e.g. BPPV), or vascular disorders (e.g. migraine, hypertension)
- Any musculoskeletal or neurological conditions that could affect balance
- Inflammatory joint disease
- Systemic conditions
- Cognitive impairment
- Taking four or more medications
- Received physiotherapy treatment for their neck disorder in the past 12 months

For those provisionally eligible for the trial, appointments will be made with an experienced physiotherapist who will be blinded to participant group allocation if accepted for the trial. The experienced physiotherapist will perform a physical examination of the neck and test sensorimotor function and balance. The physical examination includes:

1. *Tests of cervical joint position error (JPE)* [9, 13]*.* The task is to return to the starting position as accurately as possible with the blindfold. Three trials will be performed of right and left rotation and extension. An average absolute error of > 4.5° in any direction is indicative of a deficit in cervical joint position sense [34].
2. *Tandem stand test* [35]. The participants stand heel to toe with the dominant foot behind the non-dominant foot on a firm surface with eyes closed (age ≤ 45 years) or with eyes open (age > 45 years) [36]. An inability to maintain the standing position without taking a step for 30 s indicates balance impairment [36].
3. *Manual examination of the cervical spine.* The physiotherapist will palpate the cervical facet joints from C0-1 to C7-T1 bilaterally. A joint will be classified as symptomatic by a combination of pain provoked > 2/10 and the physiotherapist’s rating of at least moderately abnormal tissue resistance [37].

All participants will be provided information about the study and enrolled into the trial if they meet all eligibility criteria and voluntarily sign an informed consent statement.

### Procedure

**Fig. 1:**
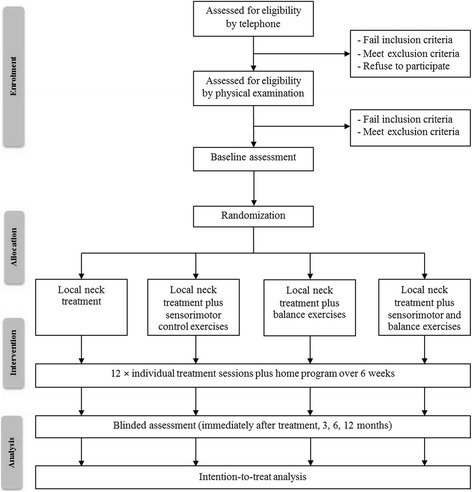
Flow diagram of the trial protocol

Eligible participants who have agreed to participate in the study will be randomly allocated to one of four treatment groups; local neck treatment (cervical mobilization and therapeutic exercise), local neck treatment combined with sensorimotor control exercises, local neck treatment combined with balance exercises, or local neck treatment combined with sensorimotor control and balance exercises. Interventions will be provided in 12 physiotherapy sessions over a 6-week intervention period (2 visits per week) [26]. Baseline and follow-up assessments (post-treatment and 3, 6 and 12 months) will be conducted at the Department of Physical Therapy, Chiang Mai University by an assessor who is blinded to treatment allocation. The participants will be requested to refrain from seeking other forms of treatment during the trial. Medication for pain may be taken, if necessary, but the participants will be asked to record the type and dose of medication in a medication diary. The flow chart of the trial is presented in Fig. 1.

### Physiotherapist training and treatment fidelity

The interventions will be provided by five physiotherapists who are experienced in the trial interventions and who have at least 5 years clinical experience in musculoskeletal physiotherapy. Each physiotherapist will deliver all intervention arms. The physiotherapists will attend a 3 h training session to enhance standardization of the interventions. The physiotherapists will be randomly assigned to each participant using computerized random numbers. Training will be provided by an experienced musculoskeletal physiotherapist and trial physiotherapists will receive a detailed procedural and treatment manual. Participant case notes will be monitored as will be selected. Treatment sessions will be audited to ensure that the physiotherapists are managing patients as per the trial treatment protocols.

### Intervention programs

Treatment will commence within one week of the baseline assessment. Each session in the intervention programs will last approximately 30-60 min depending on group allocation. The participants in each treatment group will be asked to practice their prescribed exercises once daily for 6 weeks and complete an exercise diary to monitor compliance and record adverse events. The physiotherapist will provide the elements of treatment and a home exercise program based on the initial and progressive assessment of participant’s cervical joint, muscular, and sensorimotor and balance dysfunctions (as relevant to group allocation). Description of the intervention programs are summarized in Tables 2 and 3. Exercise prescription and progression are presented in Table 4. The physiotherapist will progress the participants exercise programs to the next level once they have achieved the target of the current level.

**Table 2:** Description of the intervention programs

Intervention	Description	Time (minutes)
Local neck treatment	Cervical mobilization	10
	Specific therapeutic exercises	20
Local treatment plus sensorimotor control exercises	Cervical mobilization	10
	Specific therapeutic exercises	20
	Sensorimotor exercises	15
Local treatment plus balance exercises	Cervical mobilization	10
	Specific therapeutic exercises	20
	Balance exercises	15
Local neck treatment plus sensorimotor control and balance exercises	Cervical mobilization	10
	Specific therapeutic exercises	20
	Sensorimotor exercises	15
	Balance exercises	15

**Table 3:** Description of modalities used in the intervention programs

Modality	Description
Cervical mobilization [57]	Low-velocity passive mobilization techniques to the symptomatic cervical segments as determined by the physiotherapist’s clinical examination. Physiotherapists are free to select from what are termed passive accessory and physiological movement techniques as deemed relevant to the individual participant based on the initial and progressive reassessments.
Specific therapeutic exercises [26, 58, 59]	Cervical flexors
	(i) Train craniocervical flexor (CCF) activation and holding capacity. Participants learn the correct movement and train to hold the contraction with and without feedback in progressively more difficult inner range positions.
	(ii) Train the interaction of deep and superficial cervical flexors in movement patterning and functional tasks.
	(iii) Train co-contraction of the deep cervical flexors and extensors.
	(iv) Train strength and endurance of the cervical flexors.
	Cervical extensors
	(i) Train craniocervical extensors and rotators (head extension, head rotation < 40°) with the cervical spine in a neutral position.
	(ii) Train cervical extension to bias the cervical extensors (extend cervical spine keeping the craniocervical region in a neutral position)
	(iii) Train strength and endurance.
	Axioscapular muscles
	(i) Train scapular muscles in particular the upper/ middle/ lower trapezius and serratus anterior in both open and closed chain positions, with and without load and movement of the upper limb.
	(ii) Train correct scapular posture.
	Postural correction exercise
	(i) Train a neutral spinal posture from first treatment.
	(ii) Train scapulothoracic and cervical postures. Participants train to actively correct their posture and maintain for 10s. Practice is in sitting, standing (2-3 times an hour).
Sensorimotor exercises [7, 59]	Cervical JPS. The participants practice moving their head to points in different directions initially with eyes open, using a laser pointer mounted onto a lightweight headband. This practice will involve relocating the head back to a neutral posture or to predetermined points in range. The exercise is progressed by closing the eyes and by changing directions and ranges of movement.
	Cervical movement sense. The participants practice tracing horizontal and vertical lines on a chart on the wall focusing on accuracy and secondarily speed using a laser pointer mounted onto a lightweight headband for feedback on performance. Exercises are progressed by increasing speed and tracing more intricate patterns such as a figure of eight, zig-zag or an alphabet pattern.
	Oculomotor control exercises
	(i) Train eye follow. The participants follow a target moving from side to side and up and down while keeping their head still.
	(ii) Train gaze stability exercises. The participants perform active movements, while fixing their gaze on the target. Progressions include increasing the target’s speed, changing the participant’s position and changing visual background and focus point.
	(iii) Train eye head co-ordination exercises. The participants move their eyes and head in the same direction to focus on a target. Progressions include moving the eyes first then the head and moving eyes and head in opposite directions.
Balance exercises [7, 59]	The training starts with static balance and progresses to dynamic balance and challenging gait. The exercises will be progressed by closing the eyes, altering the support surface (i.e. a soft surface), concurrent voluntary movements, or increasing speed.

**Table 4:** Details of the exercise progression

Exercise	Level	Details	Targeted repetitions
Cervical flexors	1	Re-education of CCF movement pattern	
		Supine, knees bent	
		- Gentle and controlled nodding action facilitated with eye movement	10 reps
		Holding capacity	
		Supine, knees bent	
		- Repeated and sustained CCF progressing from 22 to 30 mmHg	10 s holds × 10 reps
	2	Interaction between the deep/superficial cervical flexors	
		Sitting	
		- Controlled head movement through range of extension and return to neutral	10 reps
		Co-contraction of the deep cervical flexors/extensors	
		Sitting	
		- Isometric cervical rotation facilitated with eye movement (left/right sides)	5 s holds × 5 reps
	3	Strength/endurance of the cervical flexors	
		Sitting	
		- Isometric CCF in a range of cervical extension	10 s holds × 10 reps
		- Lifting the head off the wall (with the chair up to 30 cm away from the wall)	10 s holds × 10 reps
		Supine	
		- Lifting the head off a pillow (2, 1 then 0 pillows as per participant’s capacity)	10 s holds × 10 reps
Cervical extensors	1	Re-education of extension movement pattern	
		Prone on elbows/four-point kneeling positions	
		- Craniocervical extension	3 sets of 5 reps
		- Craniocervical rotation (< 40°)	3 sets of 5 reps
		- Cervical extension while keeping the craniocervical region in a neutral position	3 sets of 5 reps
	2	Co-contraction of the deep cervical flexors/extensors	
		Sitting	
		- Isometric cervical rotation facilitated with eye movement (left/right sides)	5 s holds × 5reps
	3	Strength/endurance of the cervical extensors	
		Prone on elbows/four-point kneeling positions	
		- Isometric hold in range of cervical extension	10 s holds × 10 reps
		- Addition of progressive load (light weights attached to head) as per patient’s capacity	
Axioacapular control	1	Re-education of scapular movement control	
		Side lying with arm elevated 140°/sitting	
		- Passive repositioning of the scapular	10 reps
		- Active repositioning of the scapular	10 reps
		Holding capacity	
		Side lying with arm elevated 140°/sitting	
		- Active repositioning the scapular posture and isometric hold	10 s holds × 10 reps
	2	Axioscapular muscle control	
		Sitting	
		- Arm movement without load (external rotation/abduction/flexion < 30°)	10 reps
		- Arm movement without load throughout range	10 reps
		Prone on elbows/four-point kneeling position	
		- Thoracic lift (serratus anterior) and isometric hold	5 s holds × 5 reps
	3	Strength/endurance of axioscapular muscles	
		Sitting	
		- Arm movement with load (external rotation/abduction/flexion < 30°)	10 reps
		- Arm movement with load throughout the range	10 reps
		Prone	
		- Lift the shoulder off the bed and hold without arm load	10 s holds × 10 reps
		- Lift the shoulder off the bed and hold with arm load	10 s holds × 10 reps
Postural correction	1	Correction of spinal posture	
		Sitting	
		- Active upright sitting initiated with lumbo-pelvic movement	10 s holds × 10 reps
	2	Correction of spinal posture and scapular orientation	
		Sitting	
		- Actively positioning the scapular in a neutral posture while maintaining spinal posture	10 s holds × 10 reps
	3	Spinal and scapular correction plus occipital lift	
		Sitting	
		- Actively lengthen the back of the neck while maintaining spinal and scapular posture	10 s holds × 10 reps
Cervical joint position sense	1	Relocation with laser feedback	
		Sitting	
		- Head relocating to neutral position with eyes opened (vertical/horizontal)	5 reps × 3 sets
		- Head relocating to predetermined position in range with eyes opened (vertical/horizontal)	5 reps × 3 sets
	2	Relocation with laser feedback	
		Sitting	
		- Head relocating to neutral position with eyes opened (diagonal)	5 reps × 3 sets
		- Head relocating to predetermined position in range with eyes opened (diagonal)	5 reps × 3 sets
		- Head relocating to specific targets with eyes opened (all directions)	5 reps × 3 sets
	3	Relocation with laser feedback	
		Sitting	
		- Head relocating to neutral position with eyes closed (all directions)	5 reps × 3 sets
		- Head relocating to predetermined position in range with eyes closed (all directions)	5 reps × 3 sets
Cervical movement sense	1	Movement sense training with laser feedback	
		Sitting	
		- Tracing a line (vertical/horizontal)	5 reps × 3 sets
	2	Movement sense training with laser feedback	
		Sitting position	
		- Tracing an intricate pattern at a slow speed (a figure of eight/zig-zag/alphabet)	5 reps × 3 sets
	3	Movement sense training with laser feedback	
		Sitting position	
		- Tracing an intricate pattern at a fast speed (a figure of eight/zig-zag/alphabet)	5 reps × 3 sets
Oculomotor control	1	Eye follow, gaze stability and eye-head coordination	
		Sitting	
		- Eyes following a target with slow speed while keeping the head still (vertical/horizontal)	5 reps × 3 sets
		- Head moving while fixing eyes on a single spot (vertical/horizontal)	5 reps × 3 sets
		- Eyes and head moving together to the same direction (vertical/horizontal)	5 reps × 3 sets
	2	Eye follow, gaze stability and eye-head coordination	
		Sitting	
		- Eyes following a target with fast speed while keeping the head still (vertical/horizontal)	5 reps × 3 sets
		- Head moving while fixing eyes on complex targets (vertical/horizontal)	5 reps × 3 sets
		- Moving the eyes first then the head to the same direction	5 reps × 3 sets
	3	Eye follow, gaze stability and eye-head coordination	
		Sitting	
		- Eyes following a target with neck in 45° torsion (vertical/horizontal)	5 reps × 3 sets
		- Head moving while fixing eyes on a word target with complex backgrounds (vertical/horizontal)	5 reps × 3 sets
		- Eyes and head moving to the opposite direction (vertical/horizontal)	5 reps × 3 sets
Balance control	1	Static balance	
		Standing	
		- Narrow stance (firm/soft surfaces with eyes open/closed)	30 s holds × 10 reps
		- Tandem stance (firm/soft surfaces with eyes open/closed)	30 s holds × 10 reps
	2	Dynamic balance and gait	
		Standing	
		- Throwing/catching a ball while tandem stance (firm/soft surfaces)	30 s holds × 10 reps
		Walking	
		- Normal walking with fast speed (forward/backward, side way)	10 reps
		- Tandem walking (forward/backward)	10 reps
	3	Dynamic balance and gait with head movement	
		Standing	
		- Throwing/catching a ball while single leg standing (firm/soft surfaces)	30 s holds × 10 reps
		Walking	
		- Normal walking (forward/backward, side way) with head movement (left/right, up/down)	10 reps
		- Tandem walking (forward/backward) with head movement (left/right, up/down)	10 reps

### Outcome measures (Table 5)

#### Primary outcomes

##### Postural stability

A sway meter will be used to measure the extent of postural sway and displacement. The sway meter is a simple tool for assessing postural sway in individuals with impaired balance [38]. It has been shown to have good test-retest reliability (ICCs = 0.65-0.94) and good validity (r with forceplate = 0.56-0.87) [38]. The sway meter consists of a 40 cm long rod with a vertically mounted pen at its end. It will be firmly attached to the participant’s waist using a webbing strap. During the test, the pen will record participant’s sway on a millimeter graph paper fastened to the top of an adjustable-height table. Maximum displacement in anterior-posterior (APmax) and medial-lateral (MLmax) directions and total sway (number of square millimeter squares traversed by the pen) will be recorded. The postural sway will be measured in 8 conditions as follows: narrow stance (feet close together) on firm and soft surfaces with eyes open and eyes closed [39] and during a neck torsion maneuver (head turned 45° to the left and right) on firm and soft surfaces [40]. Participants will be tested barefoot and asked to stand still without talking for 30s for each condition. Participants are allowed a maximum of two additional attempts if they are unable to maintain the position for 30s. A rest period of 60s will be given between each condition.

**Table 5:** Summary of outcome measures

Primary outcome measures	Data collection instruments
Postural stability	A swaymeter
- Sway displacement	
- Sway area	
Cervical joint position error	A laser-pointer attached to a lightweight headband
Secondary outcome measures	
Gait speed	10-m walk test
Dizziness intensity	VAS (0-100 mm)
Neck pain intensity	VAS (0-100 mm)
Neck disability	NDI-TH (0-100)
Pain extent and location	iPad and custom software
Cervical range of motion	A CROM instrument
Functional ability status	PFSF (scale range 0-10)
Health-related quality of life	Thai SF-36 (scale range 0-100)
Global perceived benefit of treatment	A seven-point ordinal Likert scale (1-7)

All outcome measures will be recorded at baseline, immediately after intervention, 3, 6 and 12 months follow-ups. *VAS* Visual analogue scale, *NDI-TH* Neck disability index-Thai version, *Thai SF-36* Thai version of short form 36, *CROM* Cervical range of motion, *PFSF* The patient-specific functional scale

##### Cervical JPE

Cervical JPE will be measured using a laser-pointer attached to a lightweight headband as described by Revel et al. [13]. Participants will be seated 90 cm away from the target. They will be blindfolded and asked to perform an active movement (extension and rotation to the left and right) and return to the starting head position as accurately as possible. An absolute error between the starting and end points will be calculated in millimeters and then converted to degrees. Three repetitions of each movement direction will be undertaken and the mean value of the error will be used for analysis. This method has been shown to have good test-retest reliability (ICCs = 0.73-0.84) and high validity (r with three-dimensional ultrasound based technique = 0.95) [41].

#### Secondary outcomes

*Gait speed:* Participants will be instructed to walk barefoot over 10 m at a comfortable speed and then with head turns from side to side. The time will be measured for the intermediate 6 m to exclude acceleration and deceleration [42]. Each test will be performed twice with a 60-s rest period and the mean value used in analysis. Gait speed has been shown to be a reliable measure of functional capacity (ICC for test-retest reliability = 0.90) [43].

##### Dizziness intensity

Intensity of dizziness will be measured using a VAS [22]. Participants will be asked to indicate their average dizziness intensity over the past week by marking a horizontal 100 mm line (0 = no dizziness and 100 = worst dizziness imaginable).

##### Neck pain intensity

Neck pain intensity will be measured using a VAS. Participant will be instructed to grade their average intensity of neck pain experienced in the past week on a 0-100 mm horizontal line (0 mm = no pain and 100 mm = worst pain imaginable) [44]. The VAS has been shown to have excellent test-retest reliability (ICC = 0.97) and high validity (r with a 5-point verbal descriptive scale = 0.71-0.78) to evaluate pain perception [45, 46].

##### Neck disability

Neck disability associated with neck pain in the past week will also be measured using the NDI-TH [47]. The NDI-TH has a total of 10 sections concerning pain and activities of daily living, with a maximum score of 50. A higher score indicates greater disability. The NDI has been shown to have high validity (r with the McGill pain questionnaire = 0.69-0.70) and excellent test-retest reliability (ICC = 0.89) [48].

##### Pain extent and location

Pain drawings will be used to assess the participants’ extent and location of pain, using a digital device (iPad Air 2) and custom software [49]. Participants will be instructed to draw their pain perceived during the last week using a stylus pen on body charts with different views (frontal, dorsal, lateral right and left). They will also be asked to nominate and mark the most painful site. The type, size and colour of the pen strokes will be standardized across all participants. Custom software will be used to quantify pain extent and location. This pain drawing acquisition and analysis has been shown to be a reliable tool to evaluate the location and extent of pain in neck pain (ICC for test-retest reliability = 0.92) [49, 50].

##### Cervical range of motion

A cervical range of motion (CROM) goniometer (Performance Attainment Associates, USA) will be used to assess cervical range of motion in flexion, extension, left-right lateral flexion and left-right rotation. Participants will be seated upright and asked to actively move their neck in each direction three times. Any pain or dizziness provoked will be recorded on a 0-10 NRS. The CROM has been shown to have excellent test-retest reliability (ICCs = 0.89-0.98) and high validity (r with Fastrak motion analysis system = 0.93-0.98) [51].

##### Functional ability status

The patient-specific functional scale (PSFS) will be used to assess participants’ functional status [52]. Participants will be asked to nominate 3 to 5 activities that they are unable to do or having difficulty doing because of their neck pain. These activities will be rated on a 0-10 scale, where 0 is unable to perform the activity and 10 is able to perform the activity at same. An average of all activities scores will be used for analysis. The PSFS has been shown to have high validity (r with NDI = 0.73-0.83) and excellent test-retest reliability (ICC = 0.92) [52].

##### Health-related quality of life

The Thai version of Short Form-36 will be used to assess participants’ health-related quality of life. The instrument has been shown to have moderate to good internal consistency reliability (Cronbach’s α = 0.55-0.80) and good discriminant validity for use in a general population [53]. It contains 36 questions, divided into 8 dimensions of quality of life. These include physical functioning (10 items), role limitations due to physical health problems (4 items), social functioning (2 items), bodily pain (2 items), general mental health (5 items), vitality (4 items), role limitations due to emotional health (3 items), general health perceptions (5 items) and reported health transition (1 item). The scores for each question will be the weighted sum of the questions in each dimension. Scores range from 0 to 100, with a higher score indicating better health status.

##### Global perceived benefit of treatment

A seven-point ordinal Likert scale will be used to allow the participant to express how much they perceived a benefit from the treatment. The scale ranges from 1 (extremely dissatisfied) to 7 (extremely satisfied) [54].

### Randomization and allocation concealment

All eligible participants will be randomly allocated to one of four intervention groups: i) local neck treatment, ii) local treatment plus sensorimotor control exercises, iii) local treatment plus balance exercises, or iv) local treatment plus sensorimotor control and balance exercises. Randomization will be undertaken by an independent person with no other involvement in the trial. Random sequence will be generated by a computer permuted blocks of eight, stratified by age (≤ or > 45 years old) and dizziness (yes or no), with allocation ratio 1:1:1:1. Allocation will be concealed in sequentially numbered, sealed, opaque envelopes.

### Blinding

The baseline and follow-up assessments will be performed by an independent assessor who is blinded to treatment allocation. The physiotherapists providing the intervention will not be blinded to treatment but will be blinded to outcome assessments throughout the trial.

### Anticipated dates of trial commencement and completion

Recruitment and training of the physiotherapists was undertaken in May 2017 and recruitment of participants has commenced. All participants are expected to have completed the study by end 2019.

### Sample size

The sample size estimates are calculated based on the primary outcomes (postural stability and cervical JPE) from baseline to follow-ups. According to our previous data, we consider a sample size that gives 80% power with a 5% confidence level to detect a significant difference of 21.9 cm^2^ (SD = 12.3) for postural sway and 4.5° (SD = 3.2) for JPE. Sample size estimates were performed using both two-way ANOVA and repeated measure ANOVA (two-tailed) and the optimum number of 35 participants per group is required. To allow for 20% drop out rate, a total sample size of 168 participants (42 per group) will be recruited for this trial.

### Statistical analysis

SPSS version 17.0 or higher will be used for statistical analysis. Descriptive statistics will be used to describe demographic data and baseline characteristics and follow-ups for each treatment group. Two-way mixed ANOVA will be used to determine within-subject and between subject variables. Main effects for independent variables (local treatment/sensorimotor exercise and local treatment/balance exercise) and the intervention effects of the independent variables immediately after treatment and at 3, 6 and 12 month follow-ups will be analyzed. This allows the individual effects of sensorimotor exercise and balance exercise to be examined as well as whether there will be an additive effect of applying both sensorimotor and balance exercises in a multimodal treatment. Differences in mean change (baseline minus follow-up) will be compared between groups using baseline values of the outcomes as covariates.

Effect sizes will be calculated by taking the difference in mean changes in the primary outcomes between the intervention groups and control group (local neck treatment alone). An effect size of 0.2 will be regarded as small, 0.5 as medium and 0.8 as large [55]. Main comparative analysis for primary and secondary outcomes will be performed using an intention-to-treat approach. To address missing data, multiple imputation will be performed as a sensitivity analysis. The significance level will be set at 0.05.

## Discussion

Neck pain is a common health problem and is, along with low back pain, the world’s leading cause of years lived with disability [56]. To be effective, treatment must address not only the symptoms but also the impairments associated with neck pain. An effective treatment will help improve chances of a full recovery and prevent a recurrence of neck pain. This trial is the first to combine local neck treatment (manual therapy/therapeutic exercise) and specific approaches that target sensorimotor control and balance. The outcome of this trial will significantly facilitate informed decision making for the prevention and management of chronic neck pain.

This trial will investigate the effects of local neck treatment with and without tailored sensorimotor and balance exercise programs. The strengths of the study design are the pragmatic nature of treatment delivery towards clinical physiotherapy practices. Additionally, the exercise programs are individualized according to ongoing progress monitoring. It is expected that the findings of this trial will lead to improved clinical practice guidelines for persons with neck pain with impaired joint position sense and balance.

## Abbreviations

ANOVA: Analysis of variance
APmax: Maximum anterior-posterior
BPPV: Benign paroxysmal positional vertigo
CCF: Craniocervical flexor
CONSORT: Consolidated standards of reporting trials
CROM: Cervical range of motion
ICCs: Intraclass correlation coefficients
JPE: Joint position error
JPS: Joint position sense
MLmax: Maximum medial-lateral
NDI-TH: Neck disability index-Thai version
NRS: Numerical rating scales
PFSF: Patient-specific functional scale
SD: Standard deviation
SPSS: Statistical package for the social sciences
Thai SF-36: Thai version of short form 36
USA: United States of America
VAS: Visual analogue scale
